# A novel synonymous *TSC2* mutation in a Chinese family leads to tuberous sclerosis type 2 by disrupting Normal pre-mRNA splicing

**DOI:** 10.1016/j.jgeb.2026.100758

**Published:** 2026-06-25

**Authors:** Chuanjie Zhang, Sijun Li, Runling Leng, Min Deng, Guohua Yang

**Affiliations:** a Department of Children Health Care, Wuhan Children's Hospital (Wuhan Maternal and Child Healthcare Hospital), Tongji Medical College, Huazhong University of Science & Technology, China; b Division of Bioscience, University College London, UK; c Department of Medical Genetics, School of Basic Medical Science, Wuhan University, Wuhan 430071, China; d Department of Neurology, Renmin Hospital of Wuhan University, China; e Hubei Provincial Key Laboratory of Developmental Originated Disease, Wuhan 430071, China

**Keywords:** *TSC2*, Synonymous mutation, Splicing, Tuberous sclerosis

## Abstract

**Objective:**

To explore the molecular mechanism of tuberous sclerosis type 2 caused by *TSC2* synonymous mutations affecting normal splicing of precursor mRNA.

**Methods:**

Review and analyses clinical diagnosis and treatment process of a patient with tuberous sclerosis complex type 2, summarize the relationship between genotype and clinical phenotype. Use bioinformatics methods to analyses the effect of *TSC2* gene synonymous mutations on precursor mRNA splicing and use in vivo splicing experiments and in vitro minigene experiments to verify the possible pathogenic mechanism of *TSC2* gene synonymous mutations.

**Results:**

The proband exhibited tuberous sclerosis phenotype including epilepsy and depigmentation. A synonymous mutation in the *TSC2* gene (c.1443G > A, p.Glu481=) associated with the phenotype was identified. Bioinformatics analysis suggested that this synonymous mutation may disrupt normal pre-mRNA splicing. Both in vivo splicing assays and minigene experiments confirmed that the synonymous mutation indeed affects proper pre-mRNA splicing.

**Conclusion:**

*TSC2* c.1443G > A (p.Glu481=) synonymous mutation may be associated with tuberous sclerosis type 2 by disrupting normal pre-mRNA splicing.

## Graphical abstract

**Figure:**
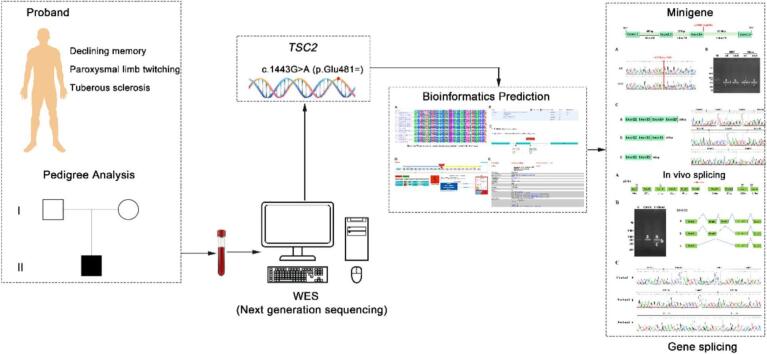

## 1 Introduction

Tuberous sclerosis complex type 2 is an autosomal dominant disorder caused by heterozygous mutations in the *TSC2* gene located on chromosome 16p13. The clinical phenotype of Tuberous sclerosis is highly heterogeneous and involve multiple organ systems including the nervous system, skin, kidneys, heart, and lungs.^1^ A hallmark feature of the disease is the development of benign hamartomas in various tissues,^2^ which can lead to complications including epilepsy, learning disabilities, behavioral problems, and renal failure.^3^, ^4^, ^5^Neurological manifestations majorly include seizures, intellectual disability, structural brain abnormalities, and autism spectrum disorder.^6^ Most patients also suffer skin lesions including hypomelanotic macules, facial angiofibroma, fibrous cephalic plaques, ungual fibromas, and Shagreen patches.^7^ Other system lesions more commonly present as renal pathologies, including angiomyolipoma and renal cysts. Due to its multisystemic nature, the clinical diagnosis of tuberous sclerosis is challenging and typically requires the presence of classic clinical features combined with confirmatory genetic testing.

The pathogenesis of tuberous sclerosis complex type 2 yet not fully elucidated. Current evid*ence suggests that the disease development is associated with mutations in the TSC1*/*TSC2* genes, while *TSC2* mutations is more prevalent and typically associated with more severe clinical manifestations. *TSC1*/*TSC2* protein complex normally functions as an inhibitor of the *mTOR* signaling pathway.^8^ Mutations in *TSC2* gene may impair the formation or function of this complex, leading to hyperactivation of *mTORC1* and subsequent multi-organ pathological changes. Previous studies have demonstrated that *TSC2* nonsense,^9^ frameshift,^10^ and missense mutations^11^ can directly or indirectly affect protein function, resulting in disease pathogenesis. López-Aranda et al. reported that *TSC2* gene deletion mutant mice exhibited impaired object location recognition, which was associated with altered IFNAR1 signaling in microglia.^12^ Conventionally, synonymous mutations were considered benign variants due to their preservation of amino acid sequences.^13^, ^14^, ^15^ However, emerging evidence indicates that synonymous mutations may contribute to disease pathogenesis through mechanisms such as altered translational folding^16^ or impaired pre-mRNA splicing.^17^ This study reports a familial case carrying a *TSC2* synonymous mutation (c.1443G > A, p.Glu481=). The proband presented with memory impairment, intermittent coma, and convulsions, with CT findings consistent indicating tuberous sclerosis. The genotype-phenotype correlation requires further validation.

Based on these findings, this study systematically reviewed the patient's clinical diagnosis and treatment process. Through comprehensive pedigree analysis and clinical phenotyping, we aimed to establish the genotype-phenotype correlation. Subsequently, employing both in vivo splicing validation and in vitro minigene assays, we investigated the impact of the *TSC2* c.1443G > A (p.Glu481=) mutation on pre-mRNA splicing. These approaches were designed to elucidate the molecular mechanism by which this synonymous mutation contributes to tuberous sclerosis pathogenesis, thereby providing novel insights for the precision diagnosis of *TSC2* and potential RNA-targeted therapeutic strategies.

## 2 Materials and methods

### 2.1 Clinical diagnosis and treatment process

The 24 years-old male patient was recruited from Renmin Hospital of Wuhan University, China with memory loss and episodic limb twitching and confusion for half a month. While routine laboratory investigations failed to identify the etiology of the seizure disorder, additional diagnostic workup including brain MRI with spectroscopy (MRS), AQP4 antibody testing, and whole-exome sequencing (WES) was done to the patient to elucidate the underlying pathological cause.

### 2.2 Diagnostic criteria

According to diagnostic criteria of International Tuberous Sclerosis Complex Diagnostic Criteria and Surveillance and Management Recommendations, Definite TSC: 2 major features or 1 major feature with 2 minor features; Possible TSC: either 1 major feature or 2 minor features; Genetic diagnosis: A pathogenic variant in *TSC1* or *TSC2* is diagnostic for TSC. A combination of the 2 major clinical features LAM and angiomyolipomas without other features does not meet criteria for a definite diagnosis.

### 2.3 Whole exon sequencing

High-throughput next-generation sequencing based on liquid-phase capture technology was employed to detect variants in exonic regions, flanking intronic regions (within 20 bp), and small insertions/deletions (<50 bp). Genomic DNA extracted from peripheral blood samples was fragmented by ultrasonication and processed for library preparation. Target regions were captured using the IDTxGen Exome Research Panel V1.0, followed by sequencing on a high-throughput sequencing platform. Quality control criteria: Target region capture and deep sequencing of disease-related genes from patient sample DNA; mean sequencing depth of 100×; Q30 ≥ 85%, Q20 ≥ 90%. Orthogonal confirmation: Sanger sequencing was performed on the patient and both parents to confirm the de novo mutation status. Variant pathogenicity was interpreted according to the ACMG standards and guidelines, population frequency data, and the HGMD disease database.

### 2.4 Bioinformatics prediction

Conservativeness analysis of the amino acids affected by the mutation was performed using PolyPhen-2 (http://genetics.bwh.harvard.edu/pph2/). The effect of the mutation on precursor mRNA splicing was analyzed using the SpliceAI (https://spliceailookup.broadinstitute.org/), Varseak (https://varseak.bio/index.php), RDDC^SC^ databases (https://rddc.tsinghua-gd.org/search-middle?to=SplitToolModel), FF database (https://www.fruitfly.org/seq_tools/splice.html), and NetGene database (https://services.healthtech.dtu.dk/services/NetGene2-2.42/). The effect of the mutation on protein expression products was analyzed using MutationTaster, and ClinVar and gnomAD were searched for pathogenic splicing variants reported at or near this position.

### 2.5 In vivo splicing analyze

Total RNA was extracted from peripheral blood samples using a commercial RNA isolation kit. Following reverse transcription into cDNA, RT-PCR amplification primers were designed based on the mutation site and gene sequence (Table 1.). PCR amplification was performed using the synthesized cDNA as template. The thermal cycling protocol consisted of an initial denaturation at 95 °C for 5 min, followed by 35 cycles of denaturation (95 °C, 30 s), annealing (57 °C, 30 s), and extension (72 °C, 90 s), with a final extension at 72 °C for 5 min. PCR products were resolved by 2% agarose gel electrophoresis, and target bands were excised and purified for subsequent Sanger sequencing.

**Table 1:** RT-PCR Primer.

Primer Name	Primer Sequence (5′-3′)
*TSC2*–817-F	TCTACAACATGTGCCACCTCATGGA
*TSC2*–1016-F	TCCTGTCCATCACCAGGCTCATCAA
*TSC2*–1783-R	GAATGTGGCTGACCAGCATCTCATA
*TSC2*–1865-R	CGCAGCAGCAACAGGAAGTCAAAG

### 2.6 Minigene splicing analyze

The recombinant vectors were transiently transfected into HeLa and 293 T cell lines using a liposome-based transfection reagent following the manufacturer's protocol. Cells were harvested 48 h post-transfection, and total RNA was extracted using a commercial RNA isolation kit according to the manufacturer's instructions. After quantifying RNA concentration, equal amounts of RNA were reverse-transcribed into cDNA.

For minigene splicing analysis, PCR amplification was performed using flanking primers (pcDNA3.1-F/pcDNA3.1-R;Table 3) specific to the pcDNA3.1 vector. The resulting transcriptional products were resolved by agarose gel electrophoresis, and individual bands were excised and purified for Sanger sequencing. Primers were designed with restriction enzyme recognition sequences (*Kpn*I, GGTACC; *Xho*I, CTCGAG) appended to their 5′ ends to facilitate directional cloning into the pcDNA3.1 vector (Table 2; lowercase letters in primer sequences denote restriction sites).

**Table 2:** Primer Sequence.

Primer Name	Primer Sequence (5′-3′)
*TSC2*–12881-F1	GTTGTGGCTACTTGGCCC
*TSC2*–13065-F2	ATCTGGGGGTGTCTCAACCC
*TSC2*–16025-R2	GGACACCCATGTCCAGGACT
*TSC2*–16443-R1	CACTGCAACCTTCACCTCCT
*TSC2*–3.1-KpnI-F⁎	AAGCTTggtaccACCTTGGACAGCC
*TSC2*-3.1-XhoI-R⁎	TAGActcgagCTTCTCGATGATGTC
*pcDNA3.1-F*	CTAGAGAACCCACTGCTTAC
*pcDNA3.1-R*	TAGAAGGCACAGTCGAGG

⁎ Restriction sites are indicated in the primer names (KpnI, XhoI). Lowercase letters in the sequences represent the terminal portions of restriction sites added for cloning purposes.

### 2.7 Statistical analysis

Statistical analysis was performed using SPSS software (version 28.0). Continuous variables were expressed as mean ± standard deviation (Mean ± SD). For normally distributed data, intergroup comparisons were conducted using independent samples *t*-test. One-way analysis of variance (ANOVA) was employed for comparisons among three or more groups. The statistical significance threshold was set at α = 0.05 (two-tailed).

## 3 Results

### 3.1 Clinical phenotypic characteristics

The proband presented with typical neurological phenotypes of tuberous sclerosis complex (TSC), including epileptic seizures and cognitive decline. The epileptic seizures manifested as limb twitching accompanied by an inability to call out, each episode lasting several minutes, with spontaneous recovery. After treatment with levetiracetam (250 mg q12h) combined with compound sodium valproate (500 mg q12h), the seizure frequency was significantly reduced. The Mini-Mental State Examination (MMSE) cognitive test score was 28 points, with deficits in memory recall, visuospatial ability, and executive function.

**Fig. 1:**
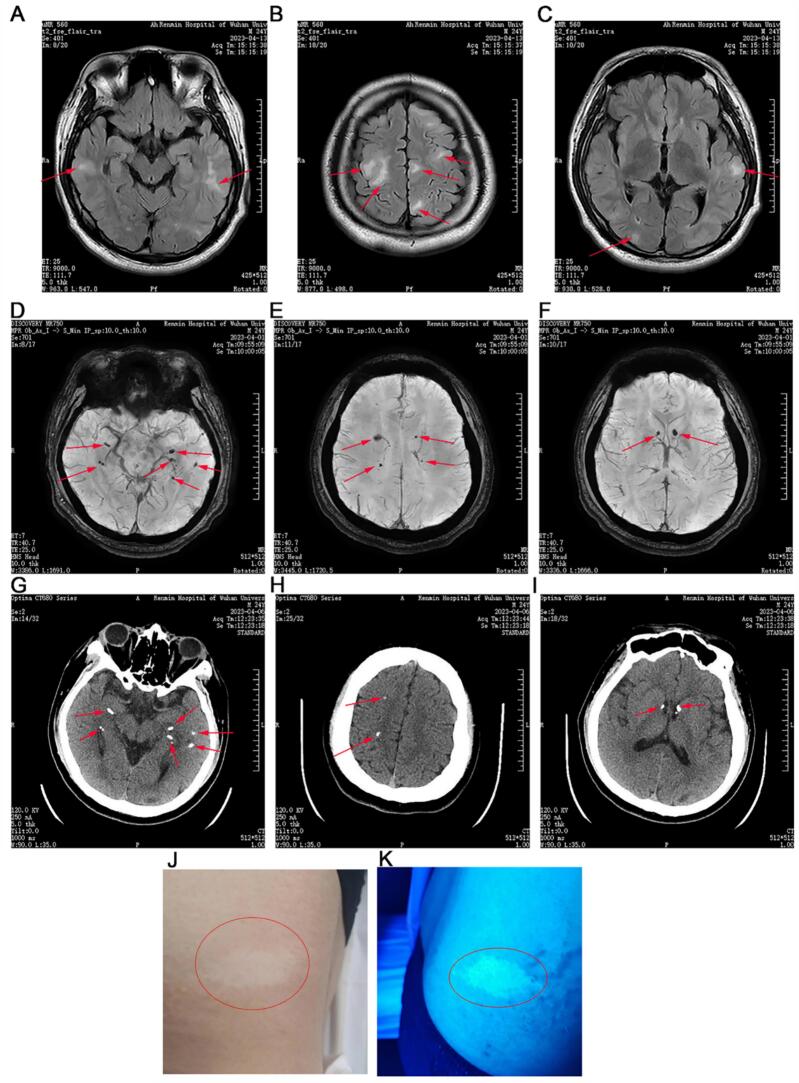
Clinical phenotypic characteristics of the proband. A-C: anti-AQP4 test result. A-C: MRI scaning results. D-F: SWI scanning results. G-I: CT scaning results. J-K: Skin examination results. The red arrow indicates the affected area. The red circle indicates a skin area with depigmentation. (For interpretation of the references to colour in this figure legend, the reader is referred to the web version of this article.)

Brain MRI revealed multiple abnormal signals in the bilateral frontal, temporal, parietal, and occipital cortices (Fig. 1A–1C), suggestive of inflammatory lesions. SWI (Fig. 1D–1F) and cranial CT (Fig. 1G–1I) showed multiple slightly low-density lesions in the bilateral frontal, temporal, parietal, and occipital cortical areas, as well as multiple calcified lesions in the bilateral frontal lobes, the left temporal lobe subcortical area, the bilateral subependymal area, and the left cerebellar hemisphere. These findings fulfill one of the major diagnostic criteria for TSC: subependymal nodules (≥2) (bilateral subependymal calcifications).

In addition to neurological phenotypes, the proband also exhibited cutaneous manifestations including hypopigmented macules. Wood's lamp examination revealed blue-white patches (Fig. 1J–1K). This fulfills another major diagnostic criterion for TSC: hypomelanotic macules (≥3, ≥5 mm in diameter).

Regarding other system assessments, cardiac, renal, hepatic, and gallbladder ultrasonography showed no rhabdomyoma, angiomyolipoma, or multiple renal cysts. Dermatological examination revealed no angiofibromas, fibrous cephalic plaques, ungual fibromas, or shagreen patches. Pulmonary imaging showed no evidence of lymphangioleiomyomatosis. Fundus examination was not performed. Dental enamel pits or intraoral fibromas were not assessed, and sclerotic bone lesions were not evaluated. Routine blood tests, coagulation function, blood biochemistry, thyroid function tests, electrocardiography, echocardiography, as well as routine and biochemical analyses of cerebrospinal fluid were all unremarkable. The central demyelinating antibody profile revealed positive anti-AQP4 antibodies, suggesting possible comorbid neuromyelitis optica spectrum disorder, which is not directly associated with TSC.

According to the International Tuberous Sclerosis Complex Consensus Conference criteria, the proband meets two major criteria; therefore, a clinical diagnosis of tuberous sclerosis complex is established without the need for minor criteria (Table 3).

**Table 3:** Diagnostic Criteria.

Diagnostic Criteria	Fulfillment status
Major Criteria	
Hypomelanotic macules (≥3; at least 5 mm diameter)	Present
Angiofibroma (≥3) or fibrous cephalic plaque	Absent
Ungual fibromas (≥2)	Absent
Shagreen patch	Absent
Multiple retinal hamartomas	Absent
Multiple cortical tubers and/or radial migration lines	Absent
Subependymal nodule (≥2)	Present
Subependymal giant cell astrocytoma	Absent
Cardiac rhabdomyoma	Absent
Lymphangiomyomatosis	Absent
Angiomyolipoma (≥2)	Absent

Minor Criteria	
“Confetti” skin lesions	Absent
Dental enamel pits (≥3)	Not assessed
Intraoral fibromas (≥2)	Not assessed
Retinal achromic patch	Not assessed
Multiple renal cysts	Absent
Nonrenal hamartomas	Absent
Sclerotic bone lesions	Not assessed

### 3.2 Whole exome sequencing results

**Fig. 2:**
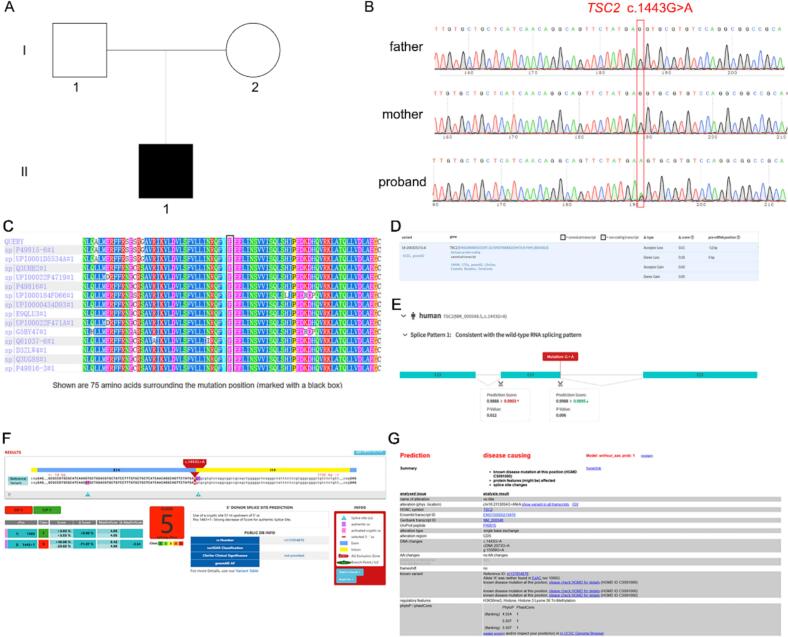
Pedigree analysis and Bioinformatics prediction results. A: Pedigree analysis results; B: Sanger sequencing results; C: Amino acid conservation analysis; D: SpliceAI prediction results; E: RDDC prediction results; F: Varseak prediction results; G: MutationTaster prediction results.

Whole-exome sequencing identified a heterozygous c.1443G > A (p.Glu481=) variant in the *TSC2* gene of the proband. Sanger sequencing results of both parents showed that they were wild-type, whereas the proband was found to carry a heterozygous mutation in the *TSC2* gene, thereby confirming that the mutation was de novo (Fig. 2A and 2B). According to the ACMG classification criteria, this variant was classified as a variant of uncertain significance (VUS) based on the following evidence: PM2_Supporting (absent in gnomAD control populations) and PP3 (predicted deleterious by multiple computational analyses including conservation profiling, evolutionary prediction, and splicing impact assessments).

### 3.3 Bioinformatics prediction results

According to Polyphen-2 analyses, mutation c.1443G > A p.Glu481 = was located at a rather conservative codon which code for glutamic acid across all 18 species. Mutation c.1443G > A p.Glu481=, located at Exon 14，belongs to “Class II mutation region” that affects shearing. 3 bioinformatics database was used to predict the splicing of such mutation. The SpliceAI algorithm predicted a reduction in the donor site confidence score (Δ = 0.18), suggesting potential splicing perturbation, Varseak analysis demonstrated a more pronounced 71.07% decrease in donor site confidence, strongly indicative of splicing disruption. In contrast, RDDC^SC^ analysis revealed no alteration in splicing patterns compared to wild-type (ΔScore 0.0015, 0.0093), showing complete concordance with normal splicing (Fig. 2). FF database and NetGene revealed no significant alteration in splicing patterns (Acceptor loss, ΔScore = 0.02, ΔScore = 0.10)(Table 4). However, the former two results could still confirm that the mutation may affect splicing. MutationTaster 2021 predicted that the c.1443G > A variant is “disease causing, protein features (might be) affected, splice site changes.” ClinVar (data accessed January 2026) classified c.1443G > A as pathogenic and has included this variant in its database. In gnomAD v4.0, the c.1443G > A variant was not detected in any population samples. However, a splicing variant p.Glu481Lys was present at this position. These supplementary data indirectly but strongly support that this variant leads to a loss of protein function.

**Table 4:** The bioinformatics prediction results of the impact of *TSC2* mutation on splicing function.

Database	c.1443G > A	ΔScore
SpliceAI	Donor loss	0.18
NetGene	Acceptor loss	0.10
Varseak	Donor loss	0.7107
FF	Acceptor loss	0.02
RDDC^sc^	Consistent with wt	0.0015, 0.0093

### 3.4 In vivo splicing analysis results

**Fig. 3:**
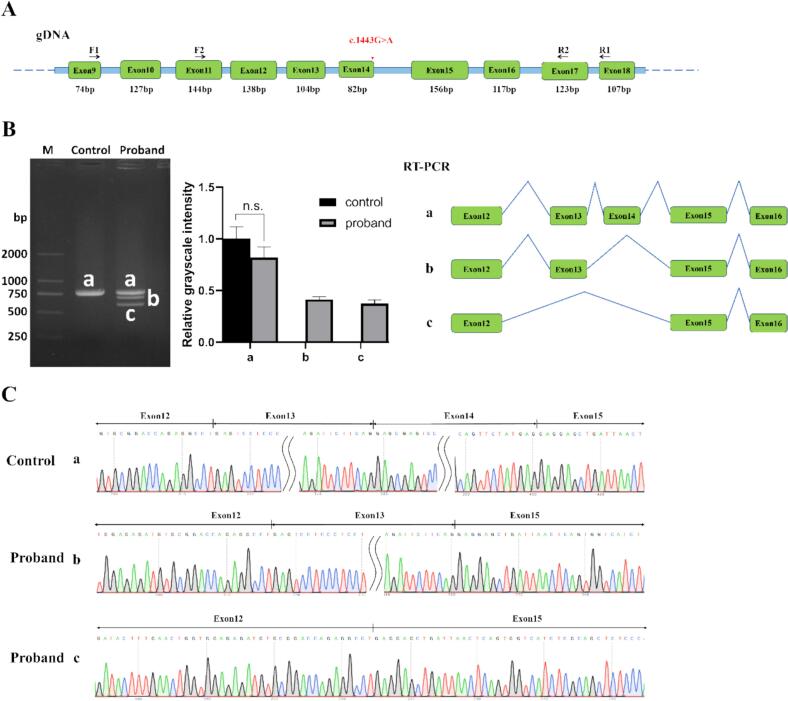
The effect of mutations on mRNA splicing A:Schematic diagram of primer design and splicing, with red arrows pointing to the mutation sites; B: Agarose gel electrophoresis of RT-PCR products, where the normal splicing bands from both normal individuals and patient samples are labeled as band a, and the abnormal splicing bands from patient samples are labeled as bands b and c; C: Sequencing results corresponding to splicing bands a, b, and c. (For interpretation of the references to colour in this figure legend, the reader is referred to the web version of this article.)

Nested PCR amplification was performed using primer pairs *TSC2*–817-F/*TSC2*–1865-R (primary round) and *TSC2*–1016-F/*TSC2*–1783-R (secondary round) on both control and patient samples. Electrophoretic analysis revealed a single 768-bp band in controls (band a), matching the expected size. In contrast, the patient sample exhibited three distinct bands: band a (similar in size to wild-type), band b (intermediate), and band c (smaller), indicating wild type individuals have only canonical splicing whereas the heterozygous patient produces both wild type and aberrant isoforms. The patient's PCR products were purified, TA-cloned, and sequenced. Sequencing results demonstrated: (1) Both control band a and patient band a represented canonical splicing (Exon12[138 bp]-Exon13[104 bp]-Exon14[82 bp]-Exon15[156 bp]-Exon16[117 bp]); (2) Patient band b showed aberrant splicing with Exon14 skipping (Exon12-Exon13-Exon15-Exon16); (3) Patient band c displayed compound exon skipping (Exon12-Exon15-Exon16) with loss of both Exon13 and Exon14 (Fig. 3).

The wild-type control individuals lacked the c.1443G > A mutation and consequently exhibited only the canonical splicing isoform. In contrast, the patient, as a heterozygous carrier of the c.1443G > A variant, demonstrated both wild-type and aberrant splicing products. Quantitative analysis results showed that the normal control sample contained only band a (100%); the patient sample was predominantly band a (50.96%), with bands b and c accounting for 25.67% and 23.37%, respectively. Aberrant splicing isoforms accounted for approximately 49.04% (band b and band c) of the total *TSC2* transcripts in the patient sample, while wild-type transcripts accounted for approximately 50.96%. There was no statistically significant difference in the levels of relative grayscale intensity of bands a between the normal control group and the patient group (*P* > 0.05) (Fig. 3). Specifically, the abnormal splicing patterns included: (1) isolated exon 14 skipping and (2) compound skipping of both exons 13 and 14, confirming the mutation's pathogenic effect on mRNA processing.

### 3.5 Mini-gene results

In both HeLa and 293 T cell lines, wild-type samples exhibited three distinct bands, designated in descending order of size as band a (∼629 bp, matching the predicted amplicon size), band b, and band c. These wild-type bands were subjected to TA cloning followed by Sanger sequencing for molecular characterization. Conversely, mutant samples in both cell lines displayed only two bands (band b and band c), which were similarly processed through TA cloning and Sanger sequencing for precise isoform identification.

**Fig. 4:**
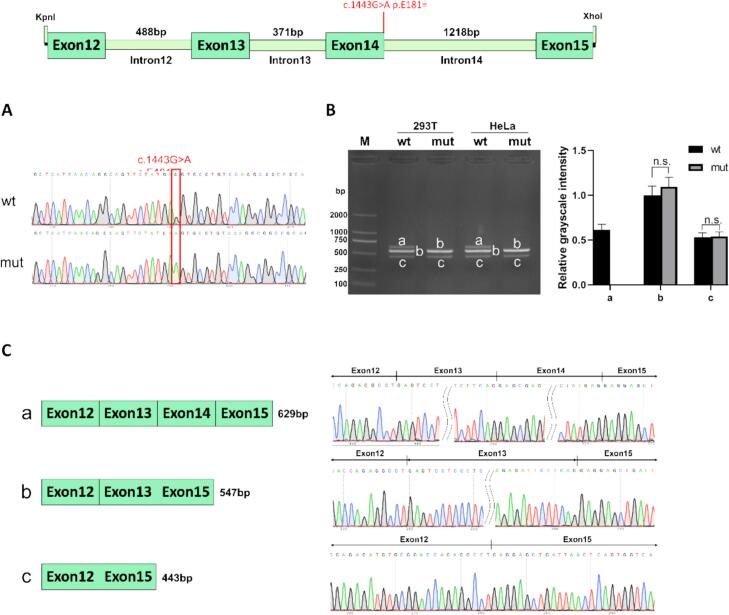
pcDNA3.1 vector detection results A: minigene construction sequencing diagram, upper panel shows wt, lower panel shows mut; B: RT-PCR transcription analysis agarose gel electrophoresis diagram; bands labeled a, b, and c in Hela and 293 T cells; C: minigene cleavage schematic diagram; cleavage bands correspond to sequencing results diagram.

Sequencing results demonstrated that wild-type band a corresponded to the canonical splicing isoform with the expected exon composition (Exon12[138 bp]-Exon13[104 bp]-Exon14[82 bp]-Exon15[156 bp]), while wild-type band b showed an aberrant splicing pattern characterized by Exon14 skipping (Exon12[138 bp]-Exon13[104 bp]-Exon15[156 bp]), and wild-type band c exhibited compound exon skipping involving both Exon13 and Exon14 (Exon12[138 bp]-Exon15[156 bp]). Notably, mutant samples displayed only abnormal splicing isoforms, with mutant band b consistently showing Exon14 skipping (Exon12[138 bp]-Exon13[104 bp]-Exon15[156 bp]) and mutant band c uniformly demonstrating the double exon skipping pattern (Exon12[138 bp]-Exon15[156 bp]), indicating a complete loss of normal splicing in the presence of the mutation. Quantitative analysis results showed that the wt group contained band a (28.62%), band b (46.67%), and band c (24.71%), with aberrant splicing isoforms accounting for 71.38% of the total TSC2 transcripts; the mut group had no band a and was predominantly composed of band b (66.99%) and band c (33.01%), with aberrant splicing isoforms accounting for 100%. There was no statistically significant difference in the levels of relative grayscale intensity of bands b and band c between the wt group and the mut group (*P* > 0.05) (Fig. 4). Minigene in vitro experimental results indicate that the mutation c.1443G > A p.E181 = affects the normal splicing of gene mRNA. After the mutation, two abnormal transcripts exist: Exon 14 skipping; Exon 13 and Exon 14 skipping.

## 4 Discussion

Tuberous sclerosis complex type 2 (*TSC2*) demonstrates marked clinical phenotypic heterogeneity, primarily characterized by multi-systemic hamartomas affecting the brain, skin, heart, kidneys, lungs, and other organs. In the present study, the proband exhibited characteristic facial angiofibromas manifesting as well-demarcated hypopigmented macules with blue-white fluorescence under Wood's lamp examination, a clinical phenotype consistent with previous reports by Boggarapu et al.^18^ in *TSC2* patients. Beyond the hallmark multi-organ hamartomatous growths, the clinical spectrum of *TSC2* additionally encompasses significant neurological impairments and renal pathologies.

In this study, the proband experienced neurological clinical phenotypes including memory impairment and epilepsy, characterized by impaired recognition of individuals, accompanied by intermittent episodes of unconsciousness and seizures lasting from several minutes to several hours, with spontaneous recovery. Following recovery, the proband exhibited slurred speech and drooling from the corners of the mouth. These findings are consistent with the typical clinical phenotype of tuberous sclerosis type 2 reported by Thiele EA et al^19^ In addition to the above two clinical phenotypes, cortical nodules and subependymal nodules are also typical clinical phenotypes of this disease. The proband's head CT showed multiple slightly hypointense lesions in the bilateral frontal, temporal, parietal, and occipital cortical regions, as well as multiple calcifications in the bilateral frontal lobes, left temporal lobe subcortical regions, bilateral subependymal regions, and left cerebellar hemisphere, suggesting tuberous sclerosis. This is consistent with the tuberous sclerosis phenotype reported by Russo for tuberous sclerosis type 2.^20^ According to the clinical diagnostic criteria for tuberous sclerosis, two major features or one major feature plus two minor features must be present, combined with genetic diagnosis, to confirm the diagnosis. In this study, the presence of two major features-facial angiofibromas and subependymal nodules-requires further genetic testing to confirm the diagnosis of tuberous sclerosis type 2.

Whole-exome sequencing of the proband identified a clinically relevant synonymous mutation (*TSC2* c.1443G > A, p.Glu481=) that further confirmed the diagnosis of tuberous sclerosis complex type 2 (*TSC2*). Although synonymous mutations are often classified as benign variants due to their preservation of amino acid sequences, the presence of characteristic *TSC2* clinical manifestations in this patient necessitates thorough investigation into the pathogenic mechanisms of this mutation. Emerging evidence suggests that synonymous mutations may contribute to disease pathogenesis through various molecular mechanisms, including altered RNA splicing,^21^ modified translational efficiency,^22^ and compromised mRNA stability.^23^

Splicing aberration would be expected to generate abnormal transcript isoforms, potentially leading to impaired or complete loss of gene function, which may represent one plausible pathogenic mechanism underlying disease development in this case. In this study, we initially employed bioinformatics approaches to assess the potential impact of this mutation on pre-mRNA splicing. The SpliceAI algorithm predicted a reduction in the donor site confidence score, Varseak analysis demonstrated a more pronounced decrease in donor site confidence. In contrast, RDDC^SC^, FF and NetGene database analysis revealed no alteration in splicing patterns, revealed that bioinformatics predictions from databases can only be used as reference, and experimental validation was still required as the primary evidence.

Functional validation of the *TSC2* c.1443G > A (p.Glu481=) variant was performed to complement the bioinformatic predictions, as computational analyses alone provide limited predictive value. In vivo splicing assays demonstrated that wild-type controls exhibited exclusively normal pre-mRNA splicing patterns, whereas heterozygous carriers of the c.1443G > A mutation produced both canonical transcripts and aberrant isoforms featuring either exon 13 skipping or compound skipping of exons 13 + 14. These findings were corroborated by parallel in vitro minigene experiments, which confirmed the mutation's disruptive effects on mRNA splicing, generating two abnormal transcript variants: ΔExon 14 and ΔExon 13 + 14. Notably, the minigene system exhibited three splicing isoforms (wild-type, ΔExon 14, and ΔExon 13 + 14) even in the wild-type construct, potentially attributable to incomplete recapitulation of native splicing regulatory elements in the artificial minigene context, leading to altered splice site recognition. The significant reduction of wild-type transcripts in this patient demonstrates the presence of a severe splicing defect, which is likely to lead to loss of function. Artificial minigenes lack a native genomic context and may carry vector-derived splicing signals, which can lead to aberrant splicing events.^24^, ^25^ The occurrence of background aberrant transcripts in wild-type minigene constructs is a known phenomenon in minigene systems; it may result from the utilization of cryptic splice sites within the cloned insert or from interactions with the vector backbone.^26^ Morak^27^ noted that minigene assays may either underestimate or overestimate the severity of certain splicing defects, while Lin^29^ further demonstrated that such discrepancies can lead to erroneous judgments regarding variant pathogenicity. For splicing analyses, the gold standard remains patient-derived RNA from the relevant tissues. In this study, in vivo RNA evidence from the patient's peripheral blood not only complemented but also strengthened the findings of the minigene assay, demonstrating that such aberrant splicing also occurs in the native environment.

The exon skipping event observed in this study may trigger nonsense-mediated mRNA decay (NMD), providing an additional molecular mechanism for haploinsufficiency. When exon skipping results in a frameshift, a randomly occurring premature termination codon (PTC) in the downstream sequence constitutes the PTC.^28^, ^29^ This alternative splicing-coupled NMD (AS-NMD) mechanism has been demonstrated to effectively “turn off” gene expression at the post-transcriptional level by switching functional transcripts into NMD substrates.^30^ If the aberrant transcripts derived from the mutant allele are eliminated via this pathway, functional proteins in the cell will be produced solely by the wild-type allele, thereby manifesting a haploinsufficiency phenotype.^31^, ^32^ Furthermore, if a small fraction of transcripts escapes NMD surveillance and are translated into truncated proteins, they may further exacerbate the phenotype through a dominant-negative effect.^33^ In the future, the CRISPRoffT database (https://ccsm.uth.edu/CRISPRoffT/)^34^ could be used to achieve in vivo TSC2 mutation construction via the CRISPR method and to observe the mutant phenotype. Additionally, single-cell RNA sequencing (scRNA-seq) analysis tools such as scLM^35^ can provide key evidence for the identification and function of TSC2 co-expressed genes.

Based on these combined bioinformatic and functional analyses, the variant was reclassified according to ACMG guidelines. The evidence now includes: (1) PVS1_Moderate (loss-of-function mechanism with exon skipping) and (2) PS2: This detection method was applied in the PS2 moderately infected group, and the test results of both parents were confirmed by Sanger sequencing. (3) PM2: Applied as PM2_Supporting by population frequency. (4) PP3: Multiple statistical methods predict that this variant will have a deleterious effect on the gene or gene product, including conservation prediction, evolutionary prediction, splicing impact. Consequently, the variant's pathogenicity classification was upgraded from variant of uncertain significance to Likely pathogenic.

In the literature results regarding the relationship between *TSC2* mutations and clinical phenotypes, seizures/epilepsy observed in Supplementary Table 1 and Supplementary Fig. 1 was the phenotype with the highest frequency of occurrence. The fundamental reason lies in the core role of the *TSC2* gene in cellular physiological function. The protein encoded by the *TSC2* gene, “Tuberin,” forms a complex with the protein “Hamartin” encoded by the *TSC1* gene.^36^ This complex is a key negative regulator of the mammalian target of rapamycin (*mTOR*) signaling pathway.^37^ This pathway is central to regulating cell growth, proliferation, and metabolism.^38^ Mutations affecting *TSC2* function led to loss of function of the *TSC1*/*TSC2* complex, which in turn causes persistent overactivation of the *mTOR* signaling pathway. In *TSC2*, pathogenic mutations that disrupt the classical splicing sites or splicing regulatory elements can prevent the spliceosome from recognizing the corresponding exons, often causing the entire exon to be removed from the mature mRNA, a phenomenon known as exon skipping.^39^ This further leads to a reduction or loss of the GAP activity of tuberin towards *Rheb*, and the accumulation of the activated *Rheb*-GTP continues, thereby constitutively activating *mTORC1*. Eventually, this converges with other loss-of-function mutations to result in excessive activation of the *mTOR* signaling pathway.^37^ This results in neuronal overgrowth, abnormal synapse formation and pruning, thereby forming structural lesions such as cortical tubers and subependymal nodules. Simultaneously, it interferes with ion channel function, neurotransmitter balance, and neural network stability, lowering the seizure threshold.^40^ Consequently, epilepsy manifests as the most core and prevalent neurological phenotype.

In summary, this study reported a family with tuberous sclerosis complex type 2 (TSC2) caused by a *TSC2* missense mutation. The proband in this family exhibited clinical phenotypes such as angiomyolipoma, subependymal nodules, and epilepsy. A *TSC2* c.1443G > A p.Glu 481 = missense mutation was identified, which was consistent with the clinical phenotypes. Bioinformatics analysis confirmed that this mutation may affect the normal splicing of pre-mRNA, and aberrant splicing bands were observed in both in vivo and in vitro minigene assays. The production of abnormal transcripts may be associated with tuberous sclerosis type 2. This study analyzed a de novo mutation in only a single proband and provided splicing experimental evidence to confirm the possible pathogenic mechanism of the de novo mutation. In the future, more *TSC2* gene mutations will be collected, and in vivo/in vitro functional validation will be performed to expand the mutation spectrum of this disease-associated gene.

## Declaration of generative AI use

The authors declare that no generative artificial intelligence (AI) tools were used in the conception, drafting, revision, or editing of this manuscript. All content, including text, analysis, and interpretation, was produced solely by the authors.

## CRediT authorship contribution statement

**Chuanjie Zhang:** Writing – original draft. **Sijun Li:** Writing – review & editing, Writing – original draft. **Runling Leng:** Formal analysis, Data curation. **Min Deng:** Supervision, Resources. **Guohua Yang:** Writing – review & editing, Supervision, Funding acquisition.

## Ethics approval and consent to participate

This study was approved by the Wuhan University Ethics Committee for Life and Medical Sciences, and informed written consent was obtained from all patients. We obtained informed consent from the patient's parents. Participants have provided written consent for the publication of clinical information and image data in this study. This study adheres to the Declaration of Helsinki, and the human data and materials used followed the Declaration of Helsinki.

## Declaration of competing interest

The authors declare that they have no known competing financial interests or personal relationships that could have appeared to influence the work reported in this paper.

## Appendix A Supplementary data

Supplementary material: Supplementary Results Mutation Spectrum
