# Supplementary material for: A novel synonymous TSC2 mutation in a Chinese family leads to tuberous sclerosis type 2 by disrupting Normal pre-mRNA splicing
Source: J Genet Eng Biotechnol. 2026 Jun 25;24(3):100758. doi: 10.1016/j.jgeb.2026.100758 (PMC13325308; doi:10.1016/j.jgeb.2026.100758)
Supplement: Supplementary file 1 — Supplementary material: Supplementary Results Mutation Spectrum [file mmc1.docx]

**Supplementary Results**

**Mutation Spectrum**

To date, the ClinVar database has reported a total of 13004 mutations in the *TSC2* gene. Among these, pathogenic mutations account for 1305 (10.1%), likely pathogenic mutations for 325 (2.5%), uncertain significance for 4303 (33.1%), likely benign for 3852 (29.6%), benign for 1722 (13.2%), conflicting classifications for 1497 (11.5%). Within the categories of pathogenic and likely pathogenic mutations, there are 177 missense mutations (10.9%), 287 nonsense mutations (17.7%), 575 frameshift mutations (35.5%), 252 splice site mutations (15.5%), 231 ncRNA (14.2%), 100UTR (6.2%). 649 single nucleotide variants (36.4%), 241 duplications (13.5%), 570 deletions (32.0%), 43 indel (2.4%), and 280 insertions (15.7%) (ClinVar 2025).

The study summarizes 40 mutations with confirmed pathogenicity, as cited in the ClinVar database, including one mutation type identified in this study (Supplementary Figure 1). The relationship between *TSC2* gene mutations and clinical phenotypes was further analyzed. The results showed that seizures or epilepsy appeared in 29 cases, accounting for 73% (29/40). Hypomelanotic macule were appeared in 24 cases, accounting for 60% (24/40). Mental retardation or developmental delay was observed in 8 cases, accounting for approximately 25%. Behavioral abnormalities or learning disabilities was reported in 4 cases, accounting for approximately 10%. Multiple organ syndromes were observed in several patients, In the nervous system phenotype, there were 29 cases of epilepsy, 10 cases of MR/DD, 8 cases of CT, 6 cases of SEN, and 4 cases of BA/LD. In the skin system phenotype, there were 24 cases of HM, 15 cases of AF/FA, 6 cases of SP, and 4 cases of UF. In the cardiac phenotype, there were 8 cases of CR. In the renal phenotype, there were 5 cases of AML and 1 case of RC. Additionally, there were 2 cases of SEGA and 1 case of LAM among other important phenotypes (Table 1). The above results indicate that the nervous system/epilepsy phenotype is the absolute core feature, suggesting that abnormal neuronal excitability is the most common and core consequence of *TSC2* mutations. Skin pigment loss spots are key diagnostic markers, as they are the earliest and most common skin manifestations of Tuberous Sclerosis Complex (TSC). Multi-system involvement demonstrates significant disease heterogeneity: mutations widely affect multiple organ systems including the nervous system, skin, heart, and kidneys, reflecting the systemic nature of TSC.


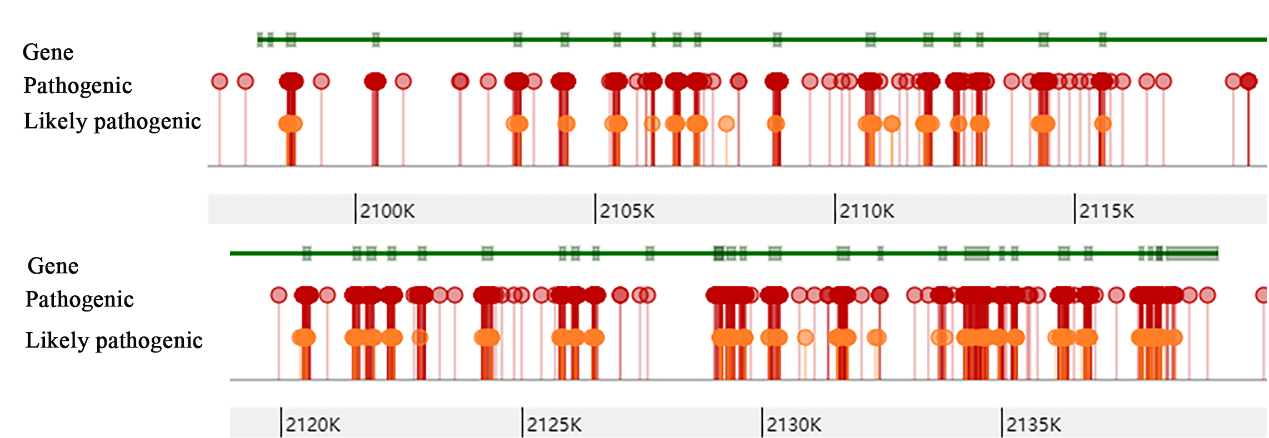


Supplementary Figure 1. Summary of mutations occurring on each region

Supplementary Table 1：Summary of *TSC2* mutations

| Mutation & Protein change | Region | Clinical significance | Clinical feature | Publication |
| --- | --- | --- | --- | --- |
| c.2742+5G>C | Intron 24 | Pathogenic | bilateral cerebellar ataxia | Fan K (2023) ^[1]^ |
| c.1864C>T (p.Arg622Trp) | Exon 16 | Pathogenic/Likely pathogenic | CR | Farach (2017) ^[2]^ |
| c.4511T>C (p.Leu1504Pro) | Exon 35 | Pathogenic | CR | He (2020) ^[3]^ |
| c.5126C>T (p.Pro1709Leu) | Exon 39 | Pathogenic | hypomelanotic macules, AF, SP, seizures, brain lesions, cognitive impairment, renal abnormalities, and cardiovascular abnormalities | Deng (2012) ^[4]^ |
| c.3598C>T (p.Arg1200Trp) | Exon 29 | Pathogenic | skin lesions, remitting epilepsy | Wentink (2012) ^[5]^ |
| c.3610G>A (p.Gly1204Arg) | Exon 29 | Likely pathogenic | AML, pulmonary lymphangioleiomyomatosis  and retinal hamartoma | Zhang R (2018) ^[6]^ |
| c.2838-122G>A | Intron 25 | Pathogenic | SEN, CT, FA, HM | Nellist M (2015) ^[7]^ |
| c.225+2T>A | Intron 3 | Pathogenic | SEGA, SEN, CT, FA, HM | Nellist M (2015) ^[7]^ |
| c.3099C>G (p.Tyr1033Ter) | Exon 16 | Pathogenic | SEN, CT, FA, HM | Nellist M (2015) ^[7]^ |
| c.2714G>A (p.Arg905Gln) | Exon 23 | Pathogenic/ Likely pathogenic | HM, focal seizures | Jansen (2006) ^[8]^ |
| c.2713C>G (p.Arg905Gly) | Exon 23 | Pathogenic | HM, focal seizures | Jansen (2006) ^[8]^ |
| c.2713C>T (p.Arg905Trp) | Exon 23 | Pathogenic | HM, focal seizures | Jansen (2006) ^[8]^ |
| c.1477C>G (p.Leu493Val) | Exon 15 | Likely pathogenic | Cortical dysplasia, SEN, HM, epilepsy, BA, cortical dysplasia | Dufner (2020) ^[9]^ |
| c.440C>A (p.Thr147Lys) | Exon 4 | Likely pathogenic | Multiple HM | Dufner (2020) ^[9]^ |
| c.839T>C (p.Met280Thr) | Exon 8 | Likely pathogenic | Epilepsy, CR, cortical dysplasia; HM, renal US abnormality, SEN, renal cell carcinoma | Dufner (2020) ^[9]^ |
| c.2747T>C (p.Leu916Pro) | Exon 23 | Pathogenic | CR, cortical dysplasia, SEN, and epilepsy | Dufner (2020) ^[9]^ |
| c.4966G>T (p.Asp1656Tyr) | Exon 38 | Pathogenic/ Likely pathogenic | epilepsy, FA, UF, renal AML, LAM | Dufner (2020) ^[9]^ |
| c.336+1G>A | Intron 3 | Pathogenic | Delayed psychomotor development, intractable myoclonic epilepsy and white macules | Yamamoto (2002) ^[10]^ |
| c.600-1G>A | Intron 5 | Pathogenic | MR, intractable epilepsy | Yamamoto (2002) ^[10]^ |
| c.2742G>A (p.Lys914=) | Exon 23 | Pathogenic/ Likely pathogenic | Epilepsy | Yamamoto (2002) ^[10]^ |
| c.1513C>T (p.Arg505Ter) | Exon 14 | Pathogenic | HM, FA, UF, SP, CT, MR/DD, BA/LD | Au KS (1998) ^[11]^ |
| c.1832G>A (p.Arg611Gln) | Exon 16 | Pathogenic | HM, FA, CT, Seizures, MR/DD, BA/LD, abnormal eye, abnormal heart | Au KS (1998) ^[11]^ |
| c.2661T>A (p.Cys887Ter) | Exon 23 | Pathogenic | HM, FA, UF, SP, CT, abnormal eye, BA/LD, kidney tumor, abnormal heart | Au KS (1998) ^[11]^ |
| c.4859A>T (p.His1620Leu) | Exon 37 | Pathogenic | HM, CT, seizures | Au KS (1998) ^[11]^ |
| c.899G>T (p.Gly300Val) | Exon 8 | Pathogenic | HM, CR, CT, seizures | Wang (2018) ^[12]^ |
| c.3099del (p.Arg1032_Tyr1033insTer) | Exon 26 | Pathogenic | AF, HM, SP, Seizures, MR | Zhao (2006) ^[13]^ |
| c.1792T>C (p.Tyr598His) | Exon 16 | Likely pathogenic | AF, HM, seizures, MR | Zhao (2006) ^[13]^ |
| c.4952A>G (p.Asn1651Ser) | Exon 37 | Pathogenic | HM, seizures, MR, cardiac tumor | Zhao (2006) ^[13]^ |
| c.5024C>T (p.Pro1675Leu) | Exon 38 | Pathogenic | AF, UF, HM, seizures ,MR, CR | Zhao (2006) ^[13]^ |
| c.3355C>T (p.Gln1119Ter) | Exon 28 | Pathogenic | Seizures, RC, hepatic AML | Zhao (2006) ^[13]^ |
| c.3099del (p.Arg1032_Tyr1033insTer) | Exon 26 | Pathogenic | AF, HM, SP, seizures, MR | Zhao (2006) ^[13]^ |
| c.5238_5255del (p.His1746_Arg1751del) | Exon 40 | Pathogenic | HM, seizures, MR | Zhao (2006) ^[13]^ |
| c.4925G>A (p.Gly1642Asp) | Exon 37 | Pathogenic | Seizures, CR | Gao (2018) ^[14]^ |
| c.1096G>T (p.Glu366Ter) | Exon 11 | Pathogenic | Seizures | Jin X (2020) ^[15]^ |
| c.2690del (p.Phe897fs) | Exon 23 | Pathogenic | Seizures, FA, renal  AML | Yu Z (2014) ^[16]^ |
| c.5138G>A (p.Arg1713His) | Exon 40 | Pathogenic/ Likely pathogenic | Seizures, HM | Fox (2017) ^[17]^ |
| c.4255_4256del (p.Gln1419fs) | Exon 37 | Pathogenic/ Likely pathogenic | Epilepsy, intellectual disability, LD, autism, HM | Farach (2023) ^[18]^ |
| c.1444-2A>G | Intron 15 | Pathogenic | Seizures, FA, white macules | Li Y (2017) ^[19]^ |
| c.496C>T (p.Gln166Ter) | Exon 5 | Pathogenic | HM, seizures, MR, CR, SEGA | Niida Y (1999) ^[20]^ |
| c.600-1G>A | Intron 5 | Pathogenic | SP, AF, HM, AML | Niida Y (1999) ^[20]^ |

AF, angiofibroma; AML: angiomyolipoma; CR: cardiac rhabdomyoma; CT: cortical tuber; FA: facial angiofibroma; HM: hypomelanotic macule; RC: renal cysts; RP: retinal phakoma; SEGA: subependymal giant cell astrocytoma; SEN: subependymal nodule; SP: shagreen patch; TE: tooth enamel defect; UF: ungual fibroma; WMA: white matter abnormalities; MR: Mental retardation; DD: developmental delay; BA: behavioral abnormalities; LD: learning disabilities.

References:

[1] Fan K, Guo Y, Song Z, Yuan L, Zheng W, Hu X, et al. The TSC2 c.2742+5G>A variant causes variable splicing changes and clinical manifestations in a family with tuberous sclerosis complex. Front Mol Neurosci. 2023, 16: 1091323.

[2] Farach LS, Gibson WT, Sparagana SP, Nellist M, Stumpel CT, Hietala M, et al. TSC2 c.1864C>T variant associated with mild cases of tuberous sclerosis complex. Am J Med Genet A. 2017, 173(3): 771-775.

[3] He S, Lv N, Bao H, Wang X, Li J. A novel TSC2 c.4511 T > C missense variant associated with tuberous sclerosis complex. BMC Med Genet. 2020, 21(1): 180.

[4] Deng X, Wu S, Deng H, Yuan L. A TSC2 recurrent variant c.5126C>T in a Han-Chinese family with tuberous sclerosis complex. Pak J Med Sci. 2025, 41(1): 263-268.

[5] Wentink M, Nellist M, Hoogeveen-Westerveld M, Zonnenberg B, van der Kolk D, van Essen T, et al. Functional characterization of the TSC2 c.3598C>T (p.R1200W) missense mutation that co-segregates with tuberous sclerosis complex in mildly affected kindreds. Clin Genet. 2012, 81(5): 453-61.

[6] Zhang R, Wang J, Wang Q, Han Y, Liu X, Bottillo I, et al. Identification of a novel TSC2 c.3610G > A, p.G1204R mutation contribute to aberrant splicing in a patient with classical tuberous sclerosis complex: a case report. BMC Med Genet. 2018, 19(1): 173.

[7] Nellist M, Brouwer RW, Kockx CE, van Veghel-Plandsoen M, Withagen-Hermans C, Prins-Bakker L, et al. Targeted Next Generation Sequencing reveals previously unidentified TSC1 and TSC2 mutations. BMC Med Genet. 2015, 16: 10.

[8] Jansen AC, Sancak O, D'Agostino MD, Badhwar A, Roberts P, Gobbi G, et al. Unusually mild tuberous sclerosis phenotype is associated with TSC2 R905Q mutation. Ann Neurol. 2006, 60(5): 528-539.

[9] Dufner Almeida LG, Nanhoe S, Zonta A, Hosseinzadeh M, Kom-Gortat R, Elfferich P, et al. Comparison of the functional and structural characteristics of rare TSC2 variants with clinical and genetic findings. Hum Mutat. 2020, 41(4): 759-773.

[10] Yamamoto T, Pipo JR, Feng JH, Takeda H, Nanba E, Ninomiya H, et al. Novel TSC1 and TSC2 mutations in Japanese patients with tuberous sclerosis complex. Brain Dev. 2002, 24(4): 227-30.

[11] Au KS, Rodriguez JA, Finch JL, Volcik KA, Roach ES, Delgado MR, et al. Germ-line mutational analysis of the TSC2 gene in 90 tuberous-sclerosis patients. Am J Hum Genet. 1998, 62(2): 286-94.

[12] Wang F, Xiong S, Wu L, Chopra M, Hu X, Wu B. A novel TSC2 missense variant associated with a variable phenotype of tuberous sclerosis complex: case report of a Chinese family. BMC Med Genet. 2018, 19(1): 90.

[13] Zhao XY, Yang S, Zhou HL, Zhu YG, Wei L, Du WH, et al. Two novel TSC2 mutations in Chinese patients with tuberous sclerosis complex and a literature review of 20 patients reported in China. Br J Dermatol. 2006, 155(5): 1070-3.

[14] Gao S, Wang Z, Xie Y. Two novel TSC2 mutations in pediatric patients with tuberous sclerosis complex: Case report. Medicine (Baltimore). 2018, 97(29): e11533.

[15] Jin X, Jin P, Yan K, Qian Y, Dong M. Genetic analysis of a mosaic case with low proportion mutation of TSC2 gene. Zhejiang Da Xue Xue Bao Yi Xue Ban. 2020, 49(5): 586-590.

[16] Yu Z, Zhang X, Guo H, Bai Y. A novel TSC2 mutation in a Chinese family with tuberous sclerosis complex. J Genet. 2014, 93(1): 169-72.

[17] Fox J, Ben-Shachar S, Uliel S, Svirsky R, Saitsu H, Matsumoto N, et al. Rare familial TSC2 gene mutation associated with atypical phenotype presentation of Tuberous Sclerosis Complex. Am J Med Genet A. 2017, 173(3): 744-748.

[18] Farach LS, Northrup H, Nellist M, van Unen L, Hillman P, Klonowska K, et al. Mild TSC phenotype and non-penetrance associated with a frameshift variant in TSC2 prompts caution in evaluating pathogenicity of frameshift variants. Gene. 2023, 877: 147566.

[19] Li Y, Cao J, Chen M, Li J, Sun Y, Zhang Y, et al. Abnormal Neural Progenitor Cells Differentiated from Induced Pluripotent Stem Cells Partially Mimicked Development of TSC2 Neurological Abnormalities. Stem Cell Reports. 2017, 8(4): 883-893.

[20] Niida Y, Lawrence-Smith N, Banwell A, Hammer E, Lewis J, Beauchamp RL, et al. Analysis of both TSC1 and TSC2 for germline mutations in 126 unrelated patients with tuberous sclerosis. Hum Mutat. 1999, 14(5): 412-22.
